# Identification of DDR1 Inhibitors from Marine Compound Library Based on Pharmacophore Model and Scaffold Hopping

**DOI:** 10.3390/ijms26031099

**Published:** 2025-01-27

**Authors:** Honghui Hu, Jiahua Tao, Lianxiang Luo

**Affiliations:** 1 The First Clinical College, Guangdong Medical University, Zhanjiang 524023, China; huhh30kaixin@gdmu.edu.cn (H.H.); jiahuatao@gdmu.edu.cn (J.T.); 2 School of Ocean and Tropical Medicine, Guangdong Medical University, Zhanjiang 524023, China

**Keywords:** DDR1 inhibitors, pharmacophore, HTVS, scaffold hopping, ADMET, molecular dynamics (MD) simulation

## Abstract

Ulcerative colitis (UC) is a chronic inflammatory condition that affects the intestines. Research has shown that reducing the activity of DDR1 can help maintain intestinal barrier function in UC, making DDR1 a promising target for treatment. However, the development of DDR1 inhibitors as drugs has been hindered by issues such as toxicity and poor binding stability. As a result, there are currently no DDR1-targeting drugs available for clinical use, highlighting the need for new inhibitors. In a recent study, a dataset of 85 DDR1 inhibitors was analyzed to identify key characteristics for effective inhibition. A pharmacophore model was constructed and validated to screen a library of marine natural products for potential DDR1 inhibitors. Through high-throughput virtual screening and precise docking, 17 promising compounds were identified from a pool of over 52,000 molecules in the marine database. To improve binding affinity and reduce potential toxicity, scaffold hopping was employed to modify the 17 compounds, resulting in the generation of 1070 new compounds. These new compounds were further evaluated through docking and ADMET analysis, leading to the identification of three compounds—39713a, 34346a, and 34419a—with superior predicted activity and drug-like properties compared to the original 17 compounds. Further analysis showed that the binding free energy values of the three candidate compounds were less than −12.200 kcal/mol, which was similar to or better than −12.377 kcal/mol of the known positive compound VU6015929, and the drug-like properties were better than those of the positive compounds. Molecular dynamics simulations were then conducted on these three candidate compounds, confirming their stable interactions with the target protein. In conclusion, compounds 39713a, 34346a, and 34419a show promise as potential DDR1 inhibitors for the treatment of ulcerative colitis.

## 1. Introduction

Inflammatory bowel disease (IBD) is a group of chronic, nonspecific inflammatory diseases of the intestine whose etiology has not yet been elucidated, particularly ulcerative colitis (UC). The main symptoms of UC are recurrent diarrhea, mucus, pus, bloody stools, and abdominal pain. The course of the disease is chronic, and the current treatment is to maintain symptom relief and promote mucosal healing (colonic lesions in the mucosa and submucosa), prevent complications, and improve the quality of life of patients [1]. Conventional treatments, including long-term use of anti-inflammatory and immunosuppressive agents, may increase the risk of serious complications such as opportunistic infections, malignancy, autoimmunity, and hepatotoxicity [1]. Consequently, the development of new therapeutic agents and strategies holds significant potential and social value.

DDR1 belongs to the DDR family, a unique group of receptor tyrosine kinases (RTKs) that are thought to play an important role in inflammatory bowel disease [2]. DDR1 is a promising therapeutic target because it is involved in the regulation of various cellular functions such as cell proliferation, differentiation, invasion, migration, and matrix remodeling, and is closely related to the occurrence and progression of many human diseases such as cancer, fibrosis, and inflammation [3]. The expression of DDR1 in intestinal epithelial cells is closely related to its function in UC, likely because DDR1 can influence epithelial cell apoptosis by regulating the expression of tight junction proteins and the integrity of the intestinal mucosal barrier [2]. DDR1 disrupts the intestinal barrier through the NF-κB p65-MLCK-p-MLC2 signaling pathway [4]. DDR1 deficiency attenuates intestinal mucosal barrier damage induced by dextran sulfate sodium (DSS)-induced colitis and reduces pro-inflammatory cytokine production [4]. The involvement of DDR1 in the pathogenesis of colitis by mediating intestinal mucosal barrier damage in UC has been demonstrated [4], making DDR1 a novel target for the treatment of intestinal inflammation.

Early DDR1 inhibitors mainly targeted the ATP-binding pocket of DDR1 kinase, but the structural similarity between the ATP-binding pockets of DDR1 kinase and other protein kinases often led to “off-target” toxicity problems [5]. Research on DDR1 inhibitors for colitis treatment has focused on their anti-inflammatory effects and their role in protecting the intestinal mucosal barrier; numerous molecules have demonstrated therapeutic efficacy in preclinical studies [4]. For instance, a team led by Mingyue Zheng at SIOPA, Chinese Academy of Sciences, designed a novel DDR1 inhibitor that exhibited favorable oral therapeutic effects in DSS-induced colitis models in mice and effectively reduced inflammation [4]. In recent years, there have been continuous innovations in the design of DDR1 inhibitors by researchers, such as some designing dual-target or multi-target inhibition [3], and some developing DDR1 inhibitors that do not target the ATP-binding pocket [5]. However, no selective small molecule inhibitors have entered clinical trials thus far due to challenges related to drug selectivity, delivery methods, and the development of drug resistance [3].

Computer-aided drug design (CADD) is a crucial tool for drug development [6]. Compared to traditional methods, CADD offers the advantages of reduced costs and accelerated processes [6]. In recent years, a number of novel inhibitors with high selectivity for DDR1 have been identified through artificial intelligence techniques such as DNA coding library screening, structure-guided optimization studies, and machine learning drug design platforms [5]. Pharmacophore-based virtual screening is an efficient drug discovery method that swiftly identifies potential drug candidates from large-scale compound libraries by utilizing pharmacophore features to predict the binding affinity of compounds to specific targets [7]. Structure-based docking is a method that can focus experimental drug screening on the most promising subset of candidate compounds [8]. ADMET refers to the absorption, distribution, metabolism, excretion, and toxicity of a drug, serving as a vital indicator for evaluating drug success and durability. Suboptimal ADMET properties are a leading cause of drug development failure. Molecular dynamics simulations enhance our understanding of drug-target interactions in complex systems, thus guiding the drug discovery and design processes [9].

Marine small molecules are a vital foundation for drug research, characterized by novel chemical structures, high biological activity, and promising success rates in drug screening. They hold potential for treating major diseases, enable targeted therapies, promote sustainable resource use, and benefit from policy support and increased investment, fostering interdisciplinary research [10]. Natural products derived from marine organisms often exhibit high bioactivity, an essential advantage for drug development, which accounts for the superior success rate of marine drug development compared to traditional methods [11]. A large number of marine-derived compounds with drug potential have been discovered, such as PLK1-PBD inhibitors [12], USP7 inhibitors [13], SLC7A11 inhibitors [14], CDK4/6 inhibitors [15], and AXL/HDAC2 inhibitors [16].

**Figure 1:**
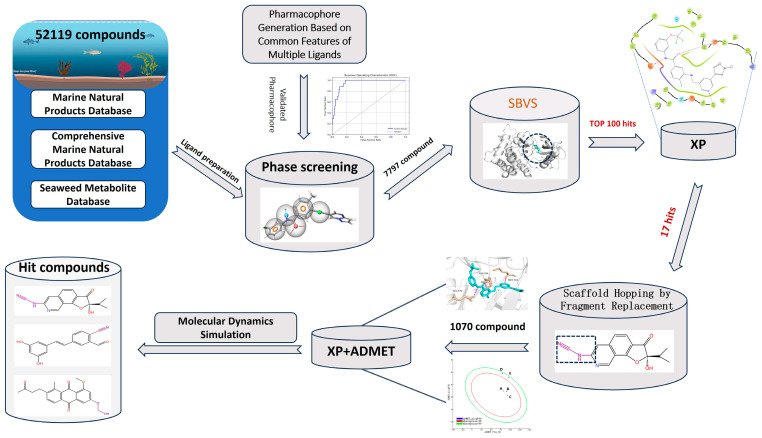
A flowchart of the strategy employed in the identification of potential inhibitors of DDR1. By constructing a pharmacophore model with multi-ligand common features to screen a compound library composed of three marine compound libraries to distinguish between active and inactive compounds, and through high-throughput virtual screening and precise docking, 17 promising potential DDR1 inhibitors were identified from the marine compound library. In order to improve the binding affinity and reduce the potential toxicity, we modified the 17 compounds by fragment substitution and re-evaluated the fragment-replaced compounds by precise docking and ADMET. Three potential DDR1 inhibitors were identified that were superior to the positive compounds, and their molecular dynamics simulations were carried out. The potential DDR1 inhibitors 39713a, 34346a, and 34419a with advantages were screened out through a comprehensive analysis of the above multiple perspectives.

In this study, in order to screen novel and active DDR1 inhibitors, we introduced the technique of fragment replacement in addition to classical drug discovery methods, including multi-ligand co-featured pharmacophore modeling, structure-based virtual screening, molecular docking, ADMET, and molecular dynamics. Thanks to the rapid development of organic synthesis methodology and computer science in recent years, such fragment replacement strategies have become more efficient and reliable, which is crucial for drug discovery. Starting from the search for DDR1 inhibitors, we sequentially completed the establishment and validation of pharmacophore models based on the common features of multiple ligands and successfully screened 17 active small molecules from 52,119 marine small molecules using pharmacophore models and structure-based virtual screening (SBVS) strategy, and optimized the small molecule structures and pharmacological properties using backbone relocation technology. Molecules 39713a, 34346a, and 34419a stood out in terms of binding efficacy and pharmacological properties and performed excellently in kinetic simulations. The idea and strategy adopted in this study are shown in Figure 1.

## 2. Results

### 2.1. Establishment and Validation of Pharmacophore Models Based on Common Features of Multiple Ligands

The pharmacophore hypothesis, which is based on the common features of multiple ligands, involves superimposing a set of ligands and extracting the essential characteristics necessary for their biological activity [17]. This approach provides a valuable technical tool for computer-aided drug design and aids in the screening of novel small-molecule inhibitors. We generated 20 distinct pharmacophore models utilizing the aforementioned data and methods, analyzing the models to reveal that DDR1 inhibitors typically contain motifs such as hydrogen-bond acceptor (A), hydrogen-bond donor (D), and aromatic ring (R). Small molecules exhibiting these three motif characteristics are more likely to be prioritized in the screening process.

Among the evaluation metrics for the 20 pharmacophores, relying solely on the Phase Hypo Score function did not guarantee evaluation accuracy. Therefore, we combined several metrics to conduct a comprehensive assessment of the pharmacophore models based on the common features of multiple ligands, ensuring that the selected models effectively distinguish between active and inactive molecules.

The ratio of active molecules in the test set to small molecules in the decoy set was utilized to calculate the enrichment factor (EF1%) for 1% of known active molecules. A higher EF1% value indicates superior pharmacophore quality. We also employed the ‘Boltzmann-enhanced Discrimination Receiver Operator’ (BEDROC) as an additional indicator for calculating EF1%. Generally, the BEDROC value fluctuates between 0 and 1; a value of 1 indicates ideal pharmacophore screening performance.

The receiver operating characteristic (ROC) curve is a model visualization tool that accurately classifies active and decoy molecules. It is widely accepted that a higher degree of convexity and skewness towards the upper left corner of the curve indicates better predictive performance. In this context, the horizontal axis represents the false positive rate (FP), while the vertical axis represents the true positive rate (TP). Any point on the curve corresponds to specific sensitivity and specificity values in a screening test. As the *x*-axis approaches zero, the predictive accuracy of the model increases. A larger *y*-axis signifies greater model sensitivity and more effective screening. When the ROC score is 1, it indicates that the pharmacophore model has excellent predictive capability, with a true positive rate of 100% and a false positive rate of 0%. Conversely, an ROC score below 0.5 suggests a lack of discriminatory ability.

The area under the accumulation curve (AUAC) reflects the diagnostic test’s magnitude; a larger area, closer to 1.0, indicates higher diagnostic accuracy, while values closer to 0.5 suggest lower accuracy. An AUAC value of 0.5 indicates no diagnostic value; however, the AUAC solely reflects the model’s overall performance and is independent of any truncation value.

**Figure 2:**
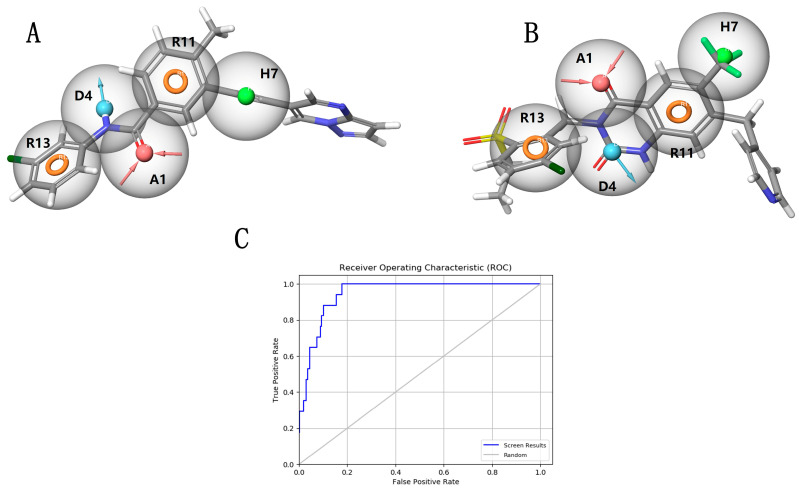
Comparative analysis of pharmacophore model interactions. (**A**) Successful alignment of pharmacophore model ADHRR_3 with active molecule 71624791; (**B**) ineffective alignment of pharmacophore model AARR_2 with inactive molecule 89884371; (**C**) receiver operating characteristic (ROC) curve for pharmacophore model ADHRR_3.

As shown in Table 1, we comprehensively evaluated the indicators of the 20 pharmacophores and selected the best model, ADHRR_3, for screening effective DDR1 inhibitors from the marine compound library. This model comprises five pharmacophore features: two aromatic rings, a hydrogen bond acceptor, a hydrogen bond donor, and a hydrophobic group. The enrichment factor for this model was 28.60, with a BEDROC value of 0.48, ROC of 0.95, and AUAC of 0.94 (Table 1, Figure 2C). These results indicate that ADHRR_3 outperformed the other 19 pharmacophore models in the comprehensive evaluation, effectively screening active small molecules from extensive small molecule data. To illustrate the discriminative ability of the pharmacophore models for active and inactive molecules, we visualized their binding pattern maps. As shown in Figure 2A,B, the small molecule 71624791 fits well with ADHRR_3, with a significant portion of it covered by pharmacophore features. In contrast, the small molecule 89884371 is poorly fitted, with almost no segments overlapping with the pharmacophore features, which suggests that the pharmacophore model possesses good discriminatory ability, further validating our choice that the ADHRR_3 pharmacophore model is suitable for screening DDR1 inhibitors in marine compound libraries.

### 2.2. Virtual Screening Based on Pharmacophore Models Based on Common Features of Multiple Ligands

Pharmacophores refer to the physicochemical features and their spatial arrangement that are essential for the molecular recognition of a ligand by a biomolecule (receptor). These “pharmacophore elements” represent the active sites involved in ligand–receptor interactions, allowing for differentiation between active and inactive small molecules. We utilized the ADHRR_3 pharmacophore to conduct pharmacophore-based virtual screening of 52,119 marine natural small molecules, which were required to exhibit at least four out of the five characteristics of the pharmacophore to qualify for screening. Ultimately, a total of 7797 active small molecules were identified for the next phase of virtual screening.

### 2.3. SBVS

Given that precision docking (XP) requires intricate intermolecular spatial morphology complementarity and energy matching, which entails significant computational resources and time, it is impractical to screen the 7797 small molecules identified by the pharmacophore model using XP. Therefore, we opted to employ HTVS to rank the affinity of these 7797 small molecules based on several parameters, including docking score, glide score, ligand efficiency, binding energy (both high and low), conformational fit at the binding position, hydrophobic interactions, hydrogen bonding, and van der Waals forces. Ultimately, the top 100 small molecules were selected for the subsequent phase of the study.

### 2.4. Molecular Docking Before Fragment Replacement

Molecular docking technology, a vital method in computer-aided drug design, is fundamentally a process of mutual recognition between two or more molecules involving spatial and energetic matching. The ligand interacts with the receptor in a manner akin to a lock and key; however, it is essential to recognize that both the receptor and ligand are flexible during molecular docking. This flexibility means that the conformation of the target protein can change throughout the binding process. Furthermore, successful docking requires not only spatial shape matching but also energy compatibility, as indicated by the change in binding free energy (ΔGbind), which determines the feasibility of their interaction [18].

**Figure 3:**
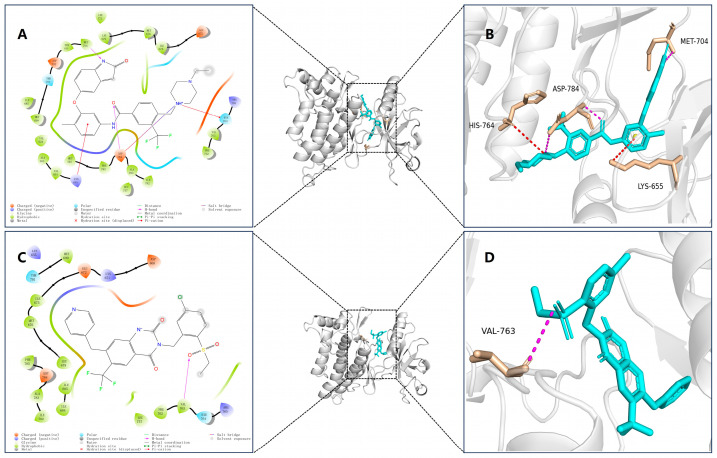
Binding pattern of DI1 and 89884371 to the protein DDR1. (**A**) Two-dimensional images of DDR1 interacting with DI1. (**B**) Three-dimensional images of DDR1 interacting with DI1. (**C**) Two-dimensional images of DDR1 interacting with 89884371. (**D**) Three-dimensional images of DDR1 interacting with 89884371.

**Figure 4:**
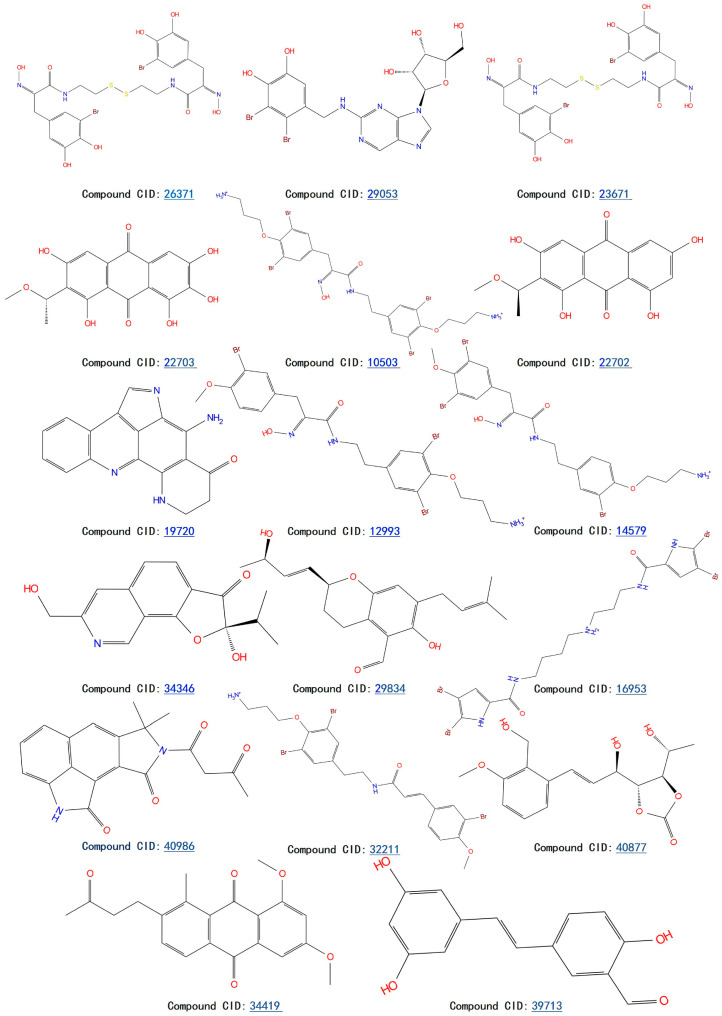
Identification of 17 marine compounds superior to positive compound VU6015929 by high-throughput virtual screening and precision docking.

Although we employed the HTVS method to screen a large number of marine small molecules rapidly, this high-speed screening sacrifices some precision. To mitigate false-positive results, we opted for the precision docking (XP) method, which more accurately predicts the binding modes of small molecules with the target DDR1. At the same time, in order to prove the reliability of the docking tool we used, we docked the DDR1 protein and the confirmed active small molecule DI1 again before the actual docking. The docking score was −12.607 kcal/mol, and the binding mode is shown in Figure 3. It shows that the target protein and the small molecule DI1 form hydrogen bond interactions and cation–π interactions, etc., similar to previous studies, and the precise binding of the active small molecules and proteins proves the reliability of the docking procedures we use. Thus, it can be used for subsequent screening and validation. We ranked the small molecules based on the calculated docking scores. To ensure accurate docking at the active site of the target, we restricted the binding pocket based on the literature, identifying Glu672, Asp702, Met704, and Asp784 as key active protein residues. The DDR1 inhibitor VU6015929, which has demonstrated activity, was used as a positive control, and positive small molecules were docked alongside 100 small molecules screened by HTVS. During precise docking, we utilized the ‘Ligand Docking’ module in Maestro 11.8, allowing each small molecule to explore up to 30 conformations to identify optimal binding poses. Ultimately, we established a cut-off docking score of −12.330 for the positive compound VU6015929 and identified 17 marine small molecules that exhibited better docking effects than this positive control. Figure 4 displays the 2D structures of these 17 small molecules for further investigation. In order to further confirm the screening ability of pharmacophores and show the differences between active and inactive molecules in the process of precise docking, we selected the negative small molecule 89884371 screened by pharmacophore for precise docking. Under the same docking conditions, the docking fraction of the negative small molecule 89884371 was −3.196 kcal/mol, which was far lower than the minimum cut-off score we set; this can be seen in the binding mode in Figure 3C,D. The negative small molecule does not bind strongly to the target protein, DDR1, and there is less interaction of forces, which further demonstrates the reliability of our screen.

### 2.5. Scaffold Hopping by Fragment Replacement

The primary goal of scaffold hopping is to enhance the physicochemical and pharmacological properties of the original drug and to develop compounds with entirely new intellectual property rights [19]. This drug design approach allows for the rational replacement of the core structure of known active compounds to create new molecules with similar three-dimensional structures to the parent compounds, potentially offering superior drug properties and increased affinities. In this study, we utilized Discovery Studio 2019 (DS) to perform scaffold hopping on 17 marine small molecules. We selected DS’s built-in fragment library, which contains 1,495,478 compound fragments that adhere to the three-fold rule (fragment molecular weight < 300, lipid–water partition coefficient, and number of hydrogen bond donors/acceptors < 3). This rule effectively limits the structural complexity of fragment molecules, ensuring that the small molecule fragments used in the substitution exhibit better water solubility. Additionally, the rule helps control the size, number of hydrogen bonds, and flexibility (number of rotatable bonds) of the fragments, allowing for potential structural modifications at later stages. During the scaffold hopping process, we analyzed regions where small molecules exhibited relatively weak binding to target proteins based on molecular docking results. Fragments that demonstrated weak interactions with the receptor were replaced, resulting in new small molecules with stronger affinities. Given the extensive number of fragments in the library, this process generated numerous new molecules. We then applied ADMET analysis, interaction force analysis, docking scores, and other evaluation criteria to screen these molecules, ultimately identifying three small molecules with optimized structures and strong drug-like properties. As shown in Table 2, we present before-and-after comparison diagrams for these three small molecules, highlighting significant improvements in their affinities and drug-like properties following scaffold hopping. This process not only enhanced drug properties, including membrane permeability, solubility, and uptake, but also improved the binding affinity of the small molecules for the target protein DDR1, offering a more effective option for screening DDR1 inhibitors.

### 2.6. Docking Analysis

**Figure 5:**
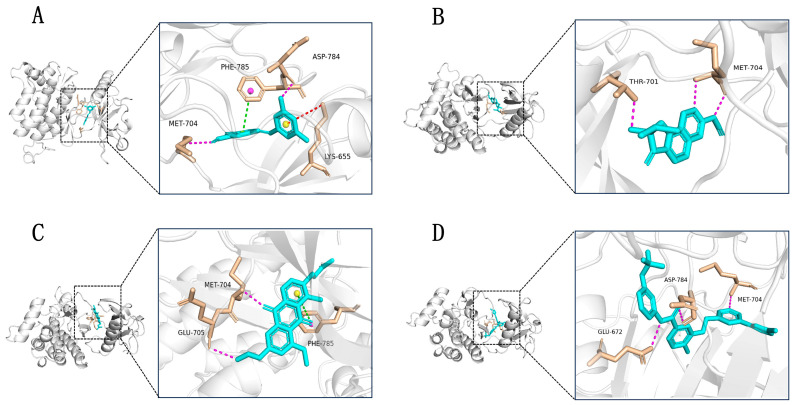
Three-dimensional visualization of binding patterns in protein–ligand complexes (hydrogen bond interactions in violet, cation–π interactions in red, π-π interactions in green). (**A**) Binding pattern of compound 39713a; (**B**) binding pattern of compound 34346a; (**C**) binding pattern of compound 34419a; (**D**) binding pattern of positive control compound VU6015929.

To further verify the reliability of the structures of the three selected small molecules (39713a, 34346a, 34419a) and the positive control (VU6015929), we conducted precision docking of these molecules using Schrödinger Suite 2019 software. The optimized structures of the three small molecules yielded results that were similar to, or better than, the positive control compound VU6015929 in precision docking. As shown in Figure 5A–D, we visualized the docking results of the three small molecules using PyMOL 2.5.0 software. The 3D structural representations indicate that compounds 39713a, 34346a, 34419a, and VU6015929, along with the positive control, formed hydrogen bonding interactions with residue Met704. Additionally, compounds 39713a and 34419a exhibited aromatic hydrogen bonding interactions with residue PHE785. In order to better highlight the activity of the optimized small molecule, we also demonstrated the binding effect of the positive small molecule VU6015929 to the compound in Figure 5D and confirmed the superior binding affinity of the three small molecules through comparison.

**Figure 6:**
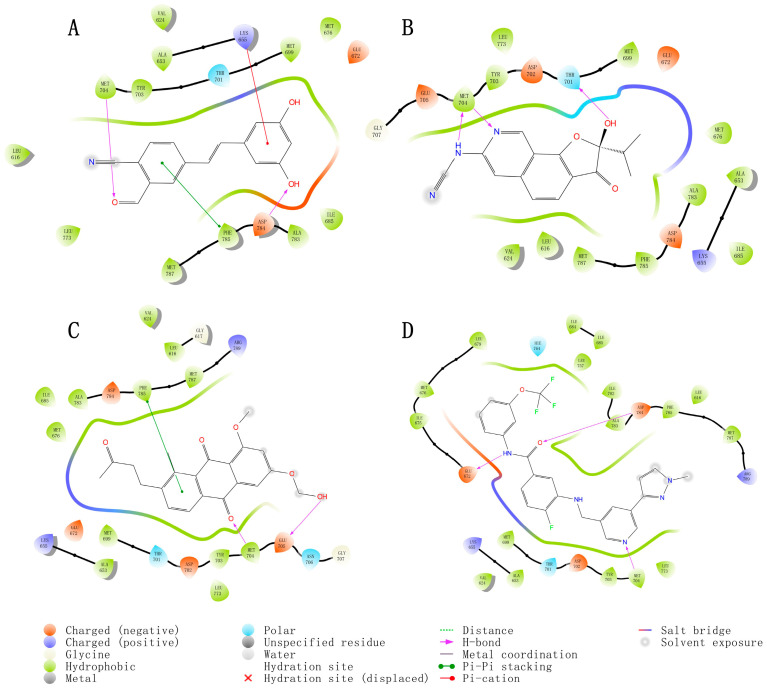
Two-dimensional images of DDR1 interacting with different compounds. (**A**) DDR1 and compound 39713a; (**B**) DDR1 and compound 34346a; (**C**) DDR1 and compound 34419a; (**D**) DDR1 and positive control compound VU6015929.

These interactions between the small molecules and target proteins were largely consistent with the docking results obtained from Maestro. In order to illustrate more clearly the conformation and interactions of small molecules within the binding pocket, we utilized the “Ligand Interaction” module in Maestro 11.8 to generate a two-dimensional diagram depicting the binding interactions between the small molecules and target proteins, as shown in Figure 6A–D. The optimized structures of these three small molecules demonstrate potential as candidate compounds.

### 2.7. ADMET

**Figure 7:**
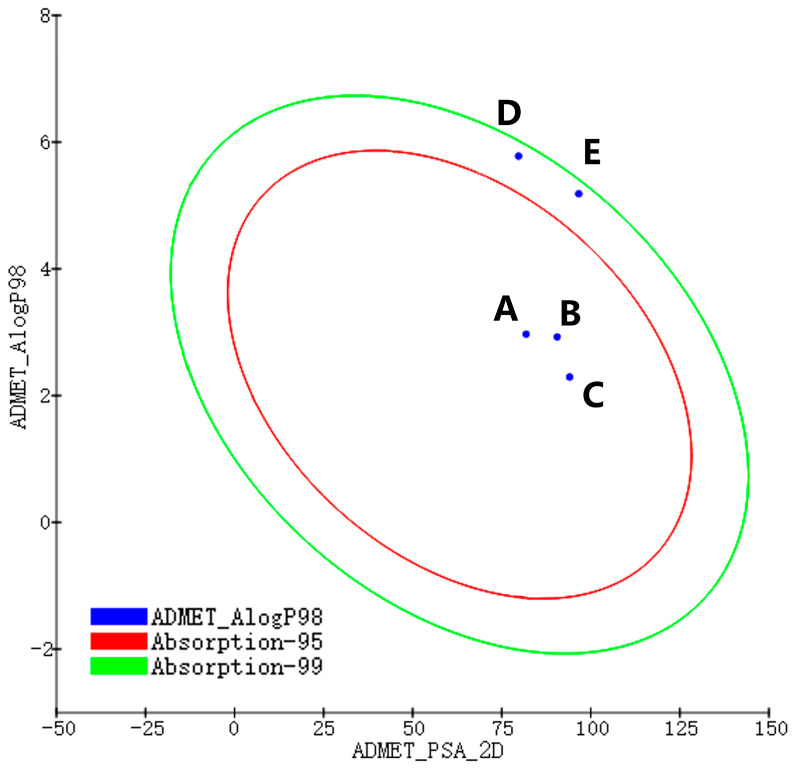
Intestinal absorption models (red ellipses represent 95% confidence intervals for the HIA model; green ellipses represent 99% confidence intervals for the HIA model. Blue dots depict the values of ADMET_PSA_2D and ADMET_AlogP98 for the three active molecules, positive control compound, and negative compound) (A: 39713-a, B: 34346-a, C: 34419-a, D: VU6015929, E: 89884371).

ADMET properties refer to the five key processes of a drug within the body: absorption, distribution, metabolism, excretion, and toxicity [20,21]. Collectively, these properties determine the ultimate efficacy and safety of a drug. In this study, Discovery Studio 2019 software was utilized to analyze the drug-like properties of small molecules following a leapfrogging approach. Three small molecules that outperformed the positive controls in terms of both affinity and drug-like properties were identified, as depicted in Figure 7. This figure illustrates two series of ellipses indicating the 95% and 99% confidence intervals for the human intestinal absorption (HIA) model. All three small molecules demonstrated favorable human intestinal absorption, with predicted values falling within the 99% confidence intervals of the HIA models. Additionally, property data regarding the ADMET profiles of the three small molecules and the positive controls are presented in Table 3. A comparison of the ADMET properties reveals that compounds 39713a, 34346a, and 34419a outperform the positive controls in terms of absorption rate, mutagenicity, and hepatotoxicity, suggesting their significant potential for future drug development. At the same time, we also explored the negative compounds, and the data showed that the drug-like properties of the negative compounds were significantly worse than those of the positive compounds, which further supported our screening results.

### 2.8. Molecular Dynamics Simulations

The potential impact of pKa values of proteins on the process of MD simulations on the functional and structural stability of proteins was analyzed before we performed molecular dynamics simulations; the results are shown in Supplementary Table S1. The results show that no protonation of amino acids is required in our pH-neutral simulation system.

**Figure 8:**
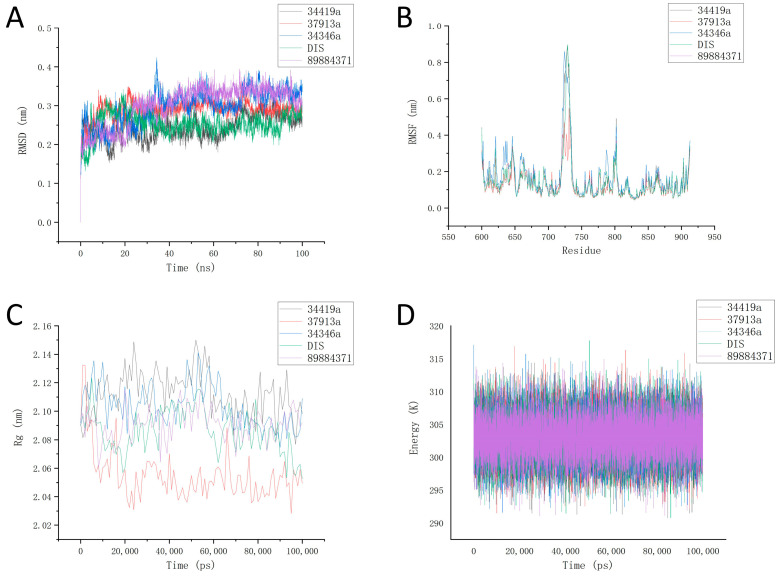
Molecular dynamics simulations of 3 ligand–protein complexes, positive control compound, and negative control compound. (**A**) RMSD values of the 3 ligand–protein complexes, positive control compound, and negative control compound over time; (**B**) schematic of the RMSF of the 3 ligand–protein complexes, positive control compound, and negative control compound; (**C**) schematic of the protein radius of gyration of the 3 ligand–protein complexes, positive control compound, and negative control compound; (**D**) overall potential energy of the 3 ligand–protein complexes, positive control compound, and negative control compound over time.

Root mean square deviation (RMSD) is a measure of the overall structural change of a protein or other molecule relative to its initial structure during a simulation. As shown in Figure 8A, during the 100 ns molecular dynamics simulation, the RMSD values of the protein–ligand complexes mainly fluctuated below 0.4, indicating that these ligand–protein complexes exhibited high stability throughout the simulation. Among them, during the simulation, our three candidate compound systems reached the equilibrium state with lower values at the 10th ns, which is closer to the positive control DI1. As shown in Supplementary Figure S1, RMSD analyses of the small molecules themselves indicate that the small molecules in the four simulated systems remained at a low level of less than 0.2 nm throughout the 100 ns simulation, reflecting the stability of the small molecules in the simulation. Among them, the RMSD of 34346a was almost always lower than that of the positive control compound DI1 in the same period, which may reflect its good binding stability, while the RMSD of 34419a and 37913a were slightly higher than that of the positive control, but also remained at an acceptable low level of less than 0.2 nm. The RMSD values of the positive compound DI1 and our three candidate compounds at simulated equilibrium are, overall, less than that of molecule 89884371, which reflects the rationality of our screening. In conclusion, all three protoprotein–ligand complexes reached a steady state during the 100 ns molecular dynamics simulation.

Root mean square fluctuation (RMSF) quantifies the range of fluctuation of each atom in a molecule. As depicted in Figure 8B, the standard deviation (SD) schematics of the five ligand complexes overlap significantly, indicating that the proteins underwent no major structural changes during the simulation, thus validating the integrity of our simulations. The positive control compound and the three candidate compounds had similar RMSF overlaps with the system composed of proteins during the simulation, reflecting the reasonable selection of candidate compounds. During RMSF analysis, we found that the negative control and our candidate compounds and positive control compounds had similar results, which may be due to the properties of the proteins themselves. Throughout the simulation, we observed that the RMSF of each protein segment remained below 0.9, reflecting low fluctuation and indicating that each residue possesses high stability.

The radius of gyration (Rg) of a protein measures the degree of structural compactness within a molecule. As shown in Figure 8C, we analyzed the radius of gyration over the entire simulation process. The results indicate that the radius of gyration fluctuated below 2.15, suggesting that the overall structure of the protein is highly compact. In the 37913a molecule–protein system, the radius of gyration of the protein was lower than that of the positive control and the other two candidate compounds for almost the entire simulation, reflecting the more compact spatial configuration of the protein. In general, the overall structure of the proteins in the three candidate compounds and the protein system remained more compact.

In order to reduce the chance bias that may be introduced when relying only on a single kinetic trajectory for the overall potential analysis of the system, in this study, three repetitions of the molecular dynamics simulation were carried out for each simulated system, and the average of the three simulations was analyzed in the overall potential analysis of the system. As presented in Figure 8D, the overall potential energy of the complex system formed by molecules 34419a, 37913a, 34346a, and DI1, along with the proteins, fluctuated at around 303 kJ/mo. In three independent iterations of the simulation, the average values of the overall potential energy of the system for 34419a, 37913a, 34346a, and DI1 are 303.1174745, 303.1019262, 303.0737617, and 303.0967909, respectively. As a comparison, the negative control small molecule 89884371 has an overall potential energy average of 303.0785536, which indicates that although this molecule was not selected by us, it still demonstrates some stability in molecular dynamics simulations. This analysis reflects structural stability from an energy standpoint.

**Figure 9:**
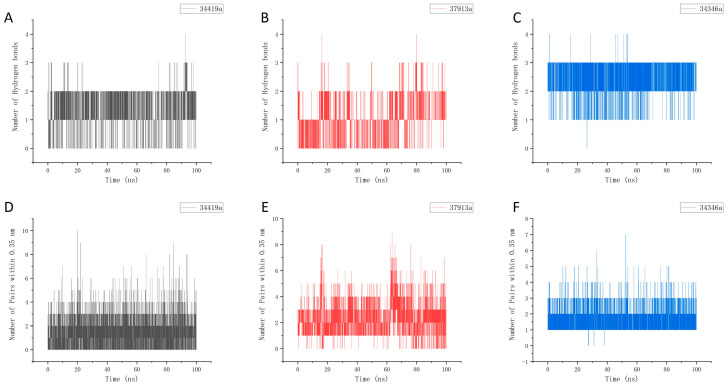
Statistical analysis of interaction numbers for three ligand–protein complexes. (**A**) Time-dependent hydrogen bond formation in the 34419a–DDR1 complex; (**B**) time-dependent hydrogen bond formation in the 37913a–DDR1 complex; (**C**) time-dependent hydrogen bond formation in the 34346a–DDR1 complex; (**D**) time-dependent interaction counts in the 34419a–DDR1 complex; (**E**) time-dependent interaction counts in the 37913a–DDR1 complex; (**F**) time-dependent interaction counts in the 34346a–DDR1 complex.

As illustrated in Figure 9, we monitored the interactions between the protein and the ligand during the simulation. Figure 9A–C depict the hydrogen bonding interactions, revealing a consistently high number of hydrogen bonds throughout the simulation, particularly for molecules 34419a and 34346a. We defined interactions based on atom distances of less than 0.35 nm, which is reflected in the overall interaction counts presented in Figure 9D–F. The results indicate that all three ligands and their corresponding proteins maintained a high number of interactions throughout the simulation, with molecule 34419a demonstrating especially notable stability.

**Figure 10:**
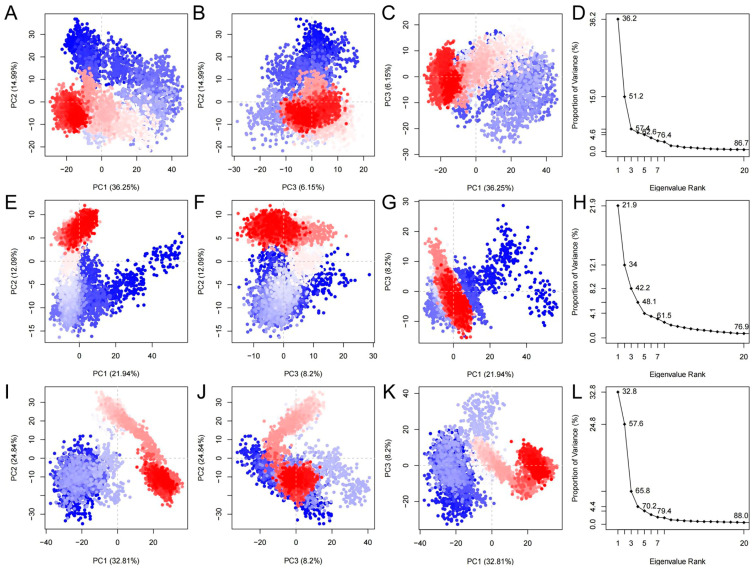
Schematic representation of principal component distributions and variance ratios of the different ligand and protein systems in the molecular dynamics simulation. Blue dots indicate early stages of the simulation, red dots indicate later stages of the simulation, and the blue to red colour gradient can help to observe the molecular trend over time during the simulation process. (**A**) Two-dimensional plot of PC1 versus PC2 for the 34419a–protein system; (**B**) 2D plot of PC2 versus PC3 for the 34419a–protein system; (**C**) 2D plot of PC1 versus PC3 for the 34419a–protein system; (**D**) plot of the eigenvalues versus the proportion of variance of the 34419a–protein system; (**E**) 2D plot of PC1 versus PC2 for the 37913a–protein system; (**F**) 2D plot of PC2 versus PC3 for the 37913a–protein system; (**G**) 2D plot of PC1 versus PC3 for the 37913a–protein system; (**H**) plot of eigenvalues versus variance scaling for the 37913a–protein system; (**I**) 2D plot of PC1 versus PC2 for the 34346a–protein system; (**J**) 2D plot of PC2 versus PC3 for the 34346a–protein system; (**K**) two-dimensional plot of PC1 versus PC3 for the 34346a–protein system; (**L**) plot of eigenvalues versus variance scaling for the 34346a–protein system.

PCA can help researchers understand the conformational changes and dynamic behavior of proteins and small molecule complexes. As shown in Figure 10, we calculated the molecular dynamics mode trajectories for PCA treatment. As shown in Figure 10D,H,L, the molecular dynamics simulations of all three ligand–protein systems can be well explained by the first three principal components. As shown in Figure 10A–C,I–K, the first three components of the 34419a–protein system and the 34346a–protein system can describe 67.39% and 65.85% of the motions of the systems, respectively. As shown in Figure 10E–G, the first three components of the 37913a–protein system can describe only 42.23% of the motion of the system, i.e., the intramolecular motions are relatively fine-grained, and there are no large motions similar to protein folding or conformational transitions.

The MM-PBSA method is a computational method used to estimate molecular binding free energy. As shown in Table 4, we calculated the binding energies of 34419a, 37913a, and 34346a binding to the target proteins. In three independent kinetic simulations for each system, the binding energy of 34419a remained below −101.801 kJ/mol and the binding energy of 34346a remained below −93.258 kJ/mol; these are very low binding energies. 37913a had a slightly higher binding energy than 34419a and 34346a, but this also remained below −72.753. Thus, the stability of the binding is reflected in terms of the binding energy.

**Figure 11:**
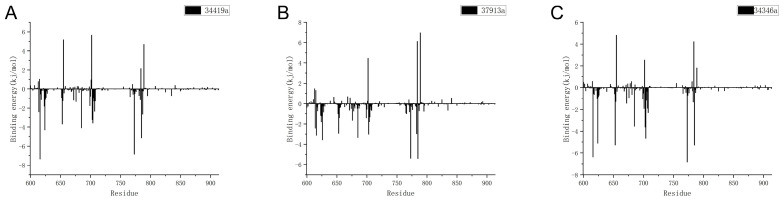
Graph of energy breakdown of protein residues at equilibrium for systems with different ligand and protein compositions. (**A**) 34419a; (**B**) 37913a; (**C**) 34346a.

We performed three independent replicates of residue decomposition binding energy calculations for each candidate compound–protein system and averaged the values for analysis. We calculated the energy breakdown of protein residues in different systems. As shown in Figure 11A–C, 34419a, 37913a, and 34346a bind to the protein, with most of the residues having a low binding energy, reflecting the stability of protein–ligand binding.

## 3. Discussion

*DDR1* is a gene that encodes a receptor tyrosine kinase [22] that is central to the initiation of signaling and plays a key role in promoting cell differentiation, proliferation, apoptosis, and migration [23,24]. Additionally, it regulates extracellular matrix homeostasis and remodeling and contributes to pathological states such as cancer, fibrosis, and inflammation [25]. DDR1 is predominantly expressed in epithelial cells across various tissues, where it not only induces the secretion of inflammatory cytokines but also amplifies its effects through stimuli such as pro-inflammatory cytokines or bacterial products [26]. In vivo inhibition of DDR1 expression has demonstrated significant therapeutic protection against DSS-induced colitis [4]. This effect may be attributed to DDR1 inhibitors blocking the activation of DDR1, thereby preventing apoptosis in intestinal epithelial cells and inhibiting the NF-κB-MLCK-P-MLC2 signaling pathway [3]. Consequently, this reduces the expression of tight junction (TJ) proteins, including ZO-1 and occludin, thereby maintaining the integrity of the intestinal barrier and decreasing the occurrence of ulcerative colitis [27]. Therefore, DDR1 may serve as a novel target for the treatment of this condition.

In this study, we assessed the current landscape of global drug and antibody development targeting DDR1 and found that the target DDR1 has a research history of more than 20 years; however, due to its high toxicity to cells, poor drug selectivity, and other reasons, researchers have been hindered from conducting more in-depth research. At the same time, similar studies in the past mainly used traditional drug screening methods. Additionally, there is no partial modification of natural compounds, which increases the limitations of the research; thus, we used traditional computer-aided drug screening technology based on current progress in computer-aided drug design to develop new research ideas for efficient screening and design of potential inhibitors of protein DDR1. We utilized computer technology and relevant software to assist in the molecular design, optimization, and screening of drugs that are highly significant for clinical treatment of ulcerative colitis [4]. The oceans, which cover more than 70% of the Earth, contain some of the richest and most diverse organisms on the planet and are a treasure trove for natural product chemistry research. Marine organisms, including sponges, corals, algae, mollusks, fish, and microorganisms, have evolved complex survival strategies under unique environments characterized by high salinity and pressure [28]. These adaptations include the production of a variety of natural compounds with unique structures and biological activities used for defense against predators, competition for space, or chemical communication with other species. Such natural compounds are increasingly becoming the focus of drug discovery and clinical trials, leading to the development of products with unique chemical structures, significant biological activity, and high medicinal value [29].

To further exploit marine resources, we integrated three databases related to marine natural products, compiling a total of 52,119 small molecules in the search for active DDR1 inhibitors. In recent years, researchers have identified several DDR1 inhibitors with varying selectivity and demonstrated their therapeutic potential in various in vivo models. However, major concerns regarding selectivity, pharmacokinetic properties, mutation resistance, and safety have been raised, resulting in the absence of selective DDR1 inhibitors in clinical studies to date. Thus, there is an urgent need to develop specific DDR1 inhibitors using various drug discovery tools. In this study, we collected 85 active DDR1 inhibitors from BindingDB and analyzed the data to develop 20 pharmacophore models based on the common features of multiple ligands. Using the bait and test sets, we calculated various metrics to evaluate the pharmacophore models and performed a comprehensive assessment. The best model, ADHRR_3, excelled across several metrics, including ROC and AUAC, indicating its superior screening performance in distinguishing active DDR1 inhibitors from a large database of small molecules. We then applied the pharmacophore model to screen an integrated marine compound library and conducted high-throughput screening and precise docking of the screened small molecules. These advanced computer screening techniques not only significantly reduce false-positive results but also accurately predict the binding patterns and modes of interaction between small molecules and their targets. This approach enabled us to select 17 marine compounds that exhibited superior properties compared to the positive compounds.

Subsequently, we performed fragment replacement on these 17 small molecules and conducted further precise docking and ADMET analyses. We compared docking scores, interaction effects, and drug-like data with those of the positive compound VU6015929, revealing that the molecules 39713a, 34346a, and 34419a demonstrated higher activity and favorable drug-like properties. Finally, the stability of these three small molecules during their interaction with target proteins was evaluated through molecular dynamics simulations. The results indicated that 39713a, 34346a, and 34419a possess ideal binding characteristics and drug-like properties, establishing them as promising candidates for the development of DDR1 kinase inhibitors.

## 4. Materials and Methods

Effective performance of the tools is essential for screening lead compounds. To enhance the credibility of the results, we utilized several well-known software packages, including Schrödinger 2019, PyMOL 2.5.0, Discovery Studio 2019, and GROMACS 2019. Specifically, the Schrödinger Suite 2019 (Schrödinger, Inc., New York, NY, USA) was employed for protein preparation, small molecule preparation, lattice generation, pharmacophore fitting and validation, pharmacophore screening, virtual screening, and molecular docking. Additionally, Discovery Studio 2019 was used for fragment replacement of small molecules, PyMOL 2.5.0 for visualizing protein–ligand complexes, and GROMACS 2019 for molecular dynamics simulation studies.

### 4.1. Protein Preparation

The crystal structure of the human DDR1 kinase domain in complex with DDR1-IN-1 was obtained from the Protein Data Bank (https://www.rcsb.org/, accessed on 29 March 2024) at a resolution of 2.2 Å (PDB Code:4CKR) [30]. This single-chain protein contains a potent inhibitor that has been extensively validated. The crystal structure was processed using the “Protein Preparation Wizard” module in Maestro 11.8 [31], which involved removing water molecules and crystalline residues (EDOs), adding missing residues and hydrogen atoms, and protonating the prepared protein structure at pH 7.0. To refine the protein structure, energy minimization was performed using a conjugate gradient method based on the OPLS3e force field for improved simulation accuracy.

### 4.2. Ligand Preparation

Studies have demonstrated that unique environmental factors, such as high salinity, high pressure, weak alkalinity, and low temperatures, contribute to the formation of many active substances in the ocean that differ from those found in terrestrial organisms. Some of these substances extracted from marine organisms exhibit antitumor, antithrombotic, and antimicrobial effects, revealing potential for screening inhibitors of DDR1 activity with novel structures. To enhance the comprehensiveness of marine compound sources, we integrated three databases related to marine natural products: (a) Marine Natural Products Database (MNPD) [32]; (b) Comprehensive Marine Natural Products Database (CMNPD) [33]; and (c) Seaweed Metabolism Database (SWMD) [34]. We aimed to utilize these valuable oceanic resources to screen for new DDR1 inhibitors. Subsequently, we employed the “LigPrep” module in Maestro 11.8 to optimize the integrated small molecules, generating 3D structures and their isomers in corresponding low-energy states. This process included (1) geometrical optimization of all small molecule structures at pH 7.0 ± 2.0, resulting in 3D structures, and (2) optimization of the 3D structures using the OPLS3e force field for energy minimization.

### 4.3. Compound Dataset Preparation

We conducted a thorough search for known DDR1 inhibitors with IC50 activity in the publicly accessible database BindingDB (https://www.bindingdb.org/rwd/bind/index.jsp, accessed on 18 April 2024). The BindingDB database contained a total of 1896 potential DDR1 inhibitors. We used IC50 values equal to 10 nM and 1000 nM as the dividing lines, defining small molecules with IC50 values less than or equal to 10 nM as active DDR1 inhibitors and small molecules with IC50 values greater than or equal to 1000 nM as inactive small molecules. We selected the active small molecules and removed duplicated data for small molecules in the database. Using these conditions, we successfully identified 85 active small molecules and 13 inactive small molecules. Additionally, we gathered 1150 bait molecules in SMILES format for the validation of pharmacophores using the online resource DUD-E (http://dude.docking.org/, accessed on 26 April 2024). Subsequently, we employed StoneMND Collector (StoneWise, Beijing, China; https://stonemind.stonewise.cn) to convert the small molecules collected in SMILES format to SDF format. These small molecules were processed using the ‘LigPrep’ module in Schrödinger, where all structures were desalted at pH 7.0 ± 2.0 using the OPLS3e force field and the Epik module, resulting in 85 small ligands with 3D structures in their corresponding low-energy states. The 85 compounds were then randomly divided into a training set and a test set in an 8:2 ratio, yielding 68 compounds for the training set and 17 for the test set. The training set was utilized to generate pharmacophore models, while the test set was employed to assess the predictive ability of these models.

### 4.4. Generation and Validation of Pharmacophore Models Based on Common Features of Multiple Ligands

Pharmacophore modeling, based on the common features of multiple ligands, highlights the spatial complementarity between small molecules and target proteins by analyzing the physicochemical characteristics necessary for ligand recognition by target protein molecules and their spatial arrangement. This approach provides guidance for small molecule screening, which was performed in this study using Schrodinger’s “PHASE” panel. In this study, the “PHASE” panel was employed to fit the collected small molecules; it has developed into a powerful platform for pharmacophore development that offers comprehensive solutions and services for life sciences. The platform includes a range of tools and procedures, from pharmacophore modeling to drug validation and screening. Based on this procedure, we classified the collected small molecules, defining those less than or equal to 10 nM as active and those greater than or equal to 1000 nM as inactive. We successfully classified the 98 collected DDR1 inhibitors into 85 active and 13 inactive molecules. Concurrently, we divided the active small molecules into experimental and validation groups for the fitting and validation of the pharmacophore. We organized these small molecules into clusters, capturing their presumed bioactive conformations and the structures contributing to their activities in three-dimensional space for structure–activity studies. This was combined with conformational analysis and molecular superposition to determine their optimal conformations and common features. We obtained a pharmacophore based on the common characteristics of these ligand molecules using the software’s built-in phase-low scoring function as an evaluation index. The characteristics of the pharmacophore were set as Acceptor (A), Donor (D), Hydrophobic (H), Negative Ion (N), Positive Ion (P), and Aromatic Ring (R), with a count of 0 to 3 and an allowable deviation of 2.0. Additionally, we required that at least 50% of the active molecules match the pharmacophore hypothesis, restricting each model to contain 4 to 6 pharmacophore feature groups and allowing a deviation from the standard of 0.5. These conditions facilitated the retrieval of as many common features of the active molecules as possible, leading to the generation of robust pharmacophore models.

### 4.5. Validation of the Model

The pharmacophore generation step often yields multiple pharmacophore models that must be validated empirically or with data. Invalid models are removed, and the retained models undergo further optimization to identify the best resulting pharmacophore model for database screening. To ensure that the pharmacophore accurately identifies active small molecules from the marine compound library, we analyzed models from a validation set composed of an active small molecule dataset (17 small molecules) combined with a decoy set (1150 small molecules). Each of the 20 generated pharmacophore models was used to screen the validation set, determining the degree of enrichment during the screening process. We employed the enrichment factor for recovering 1% of the known actives, Boltzmann-enhanced discrimination receiver operating characteristic area under the curve, receiver operating characteristic area under the curve, and area under the accumulation curve, which formed the basis for selecting the pharmacophore hypothesis.

### 4.6. Virtual Screening Based on Pharmacophore

Pharmacophore-based virtual screening is a computational chemistry approach that utilizes computer technology and bioinformatics tools to rapidly identify potentially biologically active candidate molecules from a large library of compounds, prioritizing and limiting the number of structures selected for experimental synthesis. This technique plays a crucial role in drug discovery, including the identification and optimization of lead compounds and drug design. In this study, we employed the pharmacophore model ADHRR_3 using the “Phase Ligand Screening” tool within the “Phase” module of Maestro 11.8 to virtually screen a library of 52,119 marine compounds. This approach aimed to identify small molecules with pharmacophore profiles and to isolate potential DDR1 inhibitors in the database, thereby facilitating the subsequent step of structure-based virtual screening (SBVS).

### 4.7. Structure-Based Virtual Screening

The theoretical basis of structure-based virtual screening (SBVS) is the lock-and-key theory. According to the three-dimensional structure of the target protein, small molecules are sequentially placed into the binding site of the receptor protein through molecular docking. The ligand conformation and position are continuously optimized to achieve the optimal binding state with the receptor, allowing for the determination of the binding conformation of the small molecule and the target. The binding capacity of the target and small molecule compounds is evaluated based on an affinity scoring function related to the binding energy. Compounds with more favorable binding modes and higher prediction scores are selected for subsequent bioactivity testing. To screen for active small molecules in the marine compound library, we utilized the receptor grid generator tool in the “Glide” module of Maestro 11.8 to calculate the binding pocket of the receptor DDR1 (PDB:4CKR). The van der Waals radius of the receptor atoms was scaled according to the molecular weight of the small molecules, and the compounds were docked flexibly. We employed the high-throughput virtual screening (HTVS) method to evaluate the binding ability of all compounds that successfully passed the pharmacophore screening. To enhance scoring accuracy, we allowed the conformation of the small molecules to be altered to reflect the interaction forces, enabling the “Glide” program to control conformational flexibility through extensive conformational searches, thereby eliminating inappropriate molecular conformations. We calculated the binding pocket of the receptor DDR1 (PDB:4CKR) using Maestro 11.8, scaled the van der Waals radius of the receptor atoms according to the molecular weight of the small molecules, and docked the compounds flexibly. We used the HTVS method to evaluate the binding ability of all compounds that passed the pharmacophore screening successfully. To make the scoring more accurate, we allowed the conformation of the small molecules to be altered so that the ligand’s attitude could be varied according to the interaction forces, and through these conditions, the “Glide” program can control the conformational flexibility through extensive conformational searches with the aim of eliminating inappropriate molecular conformations.

### 4.8. Molecular Docking

To further screen the DDR1 inhibitors, the 100 small molecules identified in this study underwent precision docking (XP), a computational chemistry method that simulates, at the atomic level, the binding of small molecules to biomolecular surfaces. This technique predicts possible binding modes and capacities, thereby assessing drug activity and selectivity while elucidating the fundamental biochemical processes underlying drug–protein interactions. The point that needs to be emphasized is that the good performance of molecular docking tools is necessary for structural screening and analysis of protein–ligand interaction forces. In order to undertake the molecular docking process more rigorously, before true covalent docking, the DDR1 protein (PDBID: 4CKR) is used to re-dock its eutectic ligand DI1 (PuChem cid:71664577) to the active site of the target with additional precision (XP). The analytical structure of the protein and the binding activity of the eutectic ligand have been confirmed by the researchers; by docking again and analyzing the docking fraction and binding mode, we can evaluate the performance of the molecular docking tool and decide whether to use the integration tool for the next step of screening and validation [30]. In this study, we utilized the “Receptor Grid Generation” module in Maestro 11.8 to create binding pockets at the receptor’s active site and subsequently docked the small molecules using the docking tool [35]. Asp702, Met704, and Asp784 were designated as binding sites. To enhance accuracy, we employed flexible docking and introduced the positive compound VU6015929 (a known active DDR1 inhibitor) to compare the docking scores and effects of the 100 marine compounds with the reference compound through precision docking. Small molecules exhibiting similar or superior results were selected for further fragment replacement analysis. Finally, the small molecules obtained from fragment replacement underwent additional precision docking and drug-like analysis (ADMET) to identify improved candidates for further study.

### 4.9. Scaffold Hopping by Fragment Replacement

Scaffold hopping is a widely employed drug modification strategy in both academia and industry [19]. This approach has evolved due to significant advancements in organic synthesis methodologies and computer science in recent years, enhancing the efficiency and reliability of synthesizing and rationally designing scaffold hopping analogs, which is crucial for drug discovery. The chemical structure of small molecule drugs typically comprises three components: a ring, a linker, and a side chain, with the continuous combination of the ring structure and linker referred to as the molecular backbone. We utilize visualization of the interaction forces between small molecules and receptors to identify fragments of small molecules that do not contribute to binding affinity. By judiciously replacing the core backbone of the drug, we can generate numerous new compounds with spatial structures similar to the original drug but with differing potencies. This approach provides greater opportunities to discover small molecules with favorable potency and high drug-like properties. Finally, through precise docking studies involving ADMET, we can screen for small molecules exhibiting enhanced drug-like properties and greater activity.

### 4.10. ADMET

ADMET is a comprehensive study of drug absorption, distribution, metabolism, excretion, and toxicity [36]. The evaluation of ADMET properties can effectively address the challenges associated with the poor drug characteristics of screened small molecules. This evaluation significantly enhances the success rate of drug development, reduces development costs, lowers the incidence of drug toxicity and side effects, and guides the rational use of drugs in clinical settings. Therefore, ADMET pharmacokinetic methods are essential in contemporary drug design and screening. In this study, we conducted an ADMET analysis of over one thousand small molecules generated after backbone jumping using Discovery Studio 2019. Additionally, we further screened active DDR1 inhibitors by predicting blood–brain barrier permeability (BBB), water solubility, intestinal absorption, and hepatotoxicity of the small molecules in conjunction with precise docking results.

### 4.11. Molecular Dynamics Simulation

Molecular docking methods primarily assess the theoretical binding affinity of a compound to a receptor under idealized, independent conditions; thus, favorable docking results do not fully characterize the target binding capability of a lead compound in realistic scenarios. Molecular dynamics (MD) simulations are frequently employed to monitor the stability of protein–ligand binding systems within simulated environments at specific temperatures, pressures, and salt concentrations. Firstly, we calculated the pKa of proteins using PropKa On-line (https://www.ddl.unimi.it/vegaol/propka.htm, accessed on 15 August 2024) [37] to explore the potential impact of pKa values on protein function and structural stability. Accordingly, we calculated the conformational fluctuations of complexes formed by the binding of three ligands to target proteins over a duration of 50 ns. The system’s stability was analyzed based on the conformational fluctuations of the ligands, the solvent-accessible surface area, the protein radius of gyration, and the overall potential energy of the system. Initially, mol files for ligands and PDB files for receptor proteins were generated and exported from the Discovery Studio platform. The ligand topology was constructed using the GAFF force field via the ACPYPE online server [38] (https://www.bio2byte.be/acpype/, accessed on 26 August 2024). The 2019 version of the GROMACS 2019 [39] was employed to construct topological files and perform MD calculations for proteins, utilizing the AMBER99SB-ILDN force field and the TIP3P water model. A cubic box with a radius of 1.2 nm was created to accommodate the topological system of the protein–receptor complex, which was populated with the SPC216 water model to simulate an aquatic environment. Appropriate amounts of sodium and chloride ions were added to the solvent system to neutralize the charge. Following the successful construction of the simulated system, 50,000 steps of energy minimization were conducted at a temperature of 300 K. Firstly, we performed 50,000 steps of energy minimization calculations at a simulated temperature of 300 K. Subsequently, the system was equilibrated for receptors, ligands, and solvents under constant temperature and constant volume (NVT) and constant temperature and constant pressure (NPT) conditions, with equilibration durations of 25 ps and step sizes of 25,000 steps. Van der Waals interactions during equilibration were based on cut-off values. Finally, MD simulations of the system were executed for a duration of 100 ns. Finally, we performed periodic corrections on the output trajectory files. The root mean square deviation (RMSD) and root mean square fluctuation (RMSF) of the atomic positions were analyzed, along with the radius of gyration (Rg), the total potential energy variation curve, and the number of hydrogen bonds for each system. In order to avoid chance errors caused by a single kinetic simulation on the analysis of the thermodynamic results, we performed three independent kinetic analyses for each simulated system when calculating the overall potential of the system and averaged the results of the three simulations for analysis.

We used the R language package Bio3D [40] for PCA of the simulated trajectories. Firstly, the molecular dynamics simulation trajectories were converted into DCD format, and then PCA processing and visualization of the data were performed by Bio3D.

The molecular mechanics/Poisson–Boltzmann surface area method (MM-PBSA) is widely used to calculate the free energy of receptor–ligand binding. We obtained trajectory text, topology text, and index files from molecular dynamics simulations. First, we extracted the last 90 to 100 ns of the trajectory number from molecular dynamics simulations for the calculation. We used g_MMPBSA [41,42] for MM-PBSA calculations. During the calculations, we set the dielectric constant of the solute to 2 and simulated a temperature of 300 K to calculate the van der Waals forces, the Coulomb interaction energy, the polar solvation energy, and the non-polar solvation energy. The binding energy was then calculated using the following Equation (1):

∆Gbind = Gcomplex − Gprotein − Gligand = ∆E_MM_ + ∆G_polar_ + ∆G_nonpolar_ (1)

In the above equation, Gcomplex, Gprotein, and Gligand are the free energy of the protein–ligand complex, protein free energy, and ligand free energy, respectively. ∆E_MM_ represents the energy of molecular mechanics, ∆G_polar_ represents the energy of polar solvation, and ∆G_nonpolar_ represents the energy of non-polar solvation.

For each small-molecule–protein system, we performed three independent MM-PBSA analyses and analyzed the results by averaging the results to avoid chance errors caused by single simulations of binding energy.

## 5. Conclusions

DDR1 has been identified as a potential target for treating ulcerative colitis. A pharmacophore model was created using multiple ligands and validated with 85 active small molecules. This model was used to screen a marine compound library, leading to the identification of 7797 potential compounds out of 52,119. The crystal structure of DDR1 (PDB:4CKR) was utilized for structure-based virtual screening of the 7797 compounds. A total of 17 small molecules with higher activity than the positive control compound VU6015929 were identified. These molecules were further optimized to generate 1070 new compounds. Precision docking and ADMET analysis confirmed the efficacy and favorable pharmacological properties of molecules 39713a, 34346a, and 34419a. (Binding information for these small molecules and proteins is provided in the Supplementary Materials.) Molecular dynamics simulations indicated that these compounds could serve as novel DDR1 inhibitors, presenting new treatment options for ulcerative colitis in clinical settings.

## Figures and Tables

**Table 1:** Key statistical metrics of 20 pharmacophore models.

Hypothesis	Phase Hypo Score	EF1% ^I^	BEDROC160.9 ^II^	ROC ^III^	AUAC ^IV^
ADHRR_3	1.02	28.60	0.48	0.95	0.94
ADHRR_4	1.14	28.60	0.49	0.91	0.91
ADHRR_2	0.99	5.72	0.17	0.86	0.85
ADHRR_1	1.03	17.16	0.20	0.86	0.85
ADHHR_2	1.05	17.16	0.23	0.85	0.84
DHRR_3	1.02	11.44	0.32	0.84	0.89
DHRR_2	1.00	11.44	0.23	0.84	0.89
ADHR_1	1.16	22.88	0.20	0.84	0.85
DHRR_4	0.76	22.88	0.43	0.82	0.86
ADHR_4	1.13	17.16	0.39	0.82	0.84
ADHHR_3	1.14	17.16	0.31	0.80	0.81
ADHR_2	1.06	28.60	0.57	0.77	0.82
ADHHR_1	1.09	17.16	0.27	0.74	0.76
DHRR_1	0.99	22.88	0.49	0.74	0.83
ADHR_3	1.16	17.16	0.26	0.74	0.79
AADRR_1	1.08	11.44	0.24	0.72	0.79
ADRRR_2	1.10	17.16	0.34	0.64	0.73
DRRR_1	0.97	34.32	0.57	0.47	0.72
ADRRR_1	1.08	11.44	0.31	0.39	0.63
DHHR_1	1.03	11.44	0.27	0.28	0.56

^I^ Enrichment factor for recovering 1% of the known actives; ^II^ Boltzmann-enhanced Discrimination Receiver Operator Characteristic area under the curve (alpha ¼ 160.9); ^III^ Receiver Operator Characteristic area under the curve; ^IV^ Area Under the Accumulation Curve.

**Table 2:** Results of scaffold hopping via fragment replacement (highlighted replacement structure in violet).

Name	Before Scaffold Replacement	After Scaffold Replacement	Docking Score Before Scaffold Replacement (kcal/mol)	Docking Score After Scaffold Replacement (kcal/mol)
39713-a	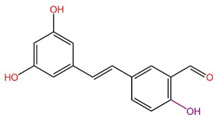	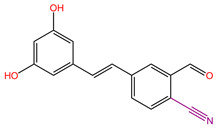	−11.757	−12.200
34346-a	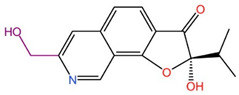	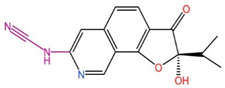	−11.048	−12.546
34419-a	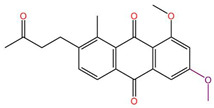	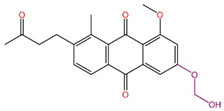	−10.121	−12.529

**Table 3:** ADMET properties of three active molecules, positive control compound, and negative compound.

Compound	Number in Figure 7	Solubility	Absorption Level	CYP2D6 Inhibit	Plasma Protein-Binding Prediction	Hepatotoxic	The Ames Test for Mutagenicity
39713-a	A	−3.065	0	−3.9461	True	−4.00616	0.674387
34346-a	B	−3.996	0	−4.32841	False	1.60824	0.68125
34419-a	C	−3.8	0	−2.37373	True	0.0071894	0.645099
VU6015929	D	−7.058	1	−12.9712	True	3.41078	0.744583
89884371	E	−6.85	1	−6.67354	True	2.4173	0.206195

**Table 4:** Energy changes calculated by the MM-PBSA method upon the interaction of candidate molecules and proteins.

Molecules	Van der Waals Energy	Electrostatic Energy	Polar Solvation Energy	SASA Energy	Binding Energy
34419a 1st	−198.518 +/− 12.397 kJ/mol	−47.493 +/− 12.177 kJ/mol	148.064 +/− 16.105 kJ/mol	−19.463 +/− 0.693 kJ/mol	−117.410 +/− 13.312 kJ/mol
34419a 2nd	−199.529 +/− 9.153 kJ/mol	−35.951 +/− 12.323 kJ/mol	143.458 +/− 20.807 kJ/mol	−19.535 +/− 0.838 kJ/mol	−111.557 +/− 17.999 kJ/mol
34419a 3rd	−175.839 +/− 15.831 kJ/mol	−36.767 +/− 18.518 kJ/mol	130.666 +/− 20.930 kJ/mol	−19.861 +/− 1.189 kJ/mol	−101.801 +/− 18.542 kJ/mol
37913a 1st	−150.872 +/− 10.482 kJ/mol	−52.345 +/− 8.556 kJ/mol	146.862 +/− 11.105 kJ/mol	−16.397 +/− 0.749 kJ/mol	−72.753 +/− 11.656 kJ/mol
37913a 2nd	−173.527 +/− 9.408 kJ/mol	−43.271 +/− 11.373 kJ/mol	136.203 +/− 11.219 kJ/mol	−16.408 +/− 0.672 kJ/mol	−97.003 +/− 9.411 kJ/mol
37913a 3rd	−165.551 +/− 10.524 kJ/mol	−44.290 +/− 8.345 kJ/mol	143.429 +/− 11.033 kJ/mol	−16.273 +/− 0.874 kJ/mol	−82.685 +/− 11.727 kJ/mol
34346a 1st	−172.099 +/− 9.348 kJ/mol	−43.435 +/− 6.575 kJ/mol	105.458 +/− 6.775 kJ/mol	−15.873 +/− 0.603 kJ/mol	−125.949 +/− 7.963 kJ/mol
34346a 2nd	−163.694 +/− 9.085 kJ/mol	−38.027 +/− 5.967 kJ/mol	118.349 +/− 7.876 kJ/mol	−15.907 +/− 0.655 kJ/mol	−99.280 +/− 9.387 kJ/mol
34346a 3rd	−159.662 +/− 10.797 kJ/mol	−38.070 +/− 6.593 kJ/mol	120.221 +/− 13.216 kJ/mol	−15.747 +/− 0.739 kJ/mol	−93.258 +/− 11.599 kJ/mol

## Data Availability

The original data presented in the study are included in the article; further inquiries can be directed to the corresponding author.

## Supplementary Materials

The following supporting information can be downloaded at: https://www.mdpi.com/article/10.3390/ijms26031099/s1.

## References

[1] Gajendran M., Loganathan P., Jimenez G., Catinella A.P., Ng N., Umapathy C., Ziade N., Hashash J.G. (2019). A Comprehensive Review and Update on Ulcerative Colitis. Disease-a-Month.

[2] Tan X., Li C., Yang R., Zhao S., Li F., Li X., Chen L., Wan X., Liu X., Yang T. (2022). Discovery of Pyrazolo[3,4-d]Pyridazinone Derivatives as Selective DDR1 Inhibitors via Deep Learning Based Design, Synthesis, and Biological Evaluation. J. Med. Chem..

[3] Liu M., Zhang J., Li X., Wang Y. (2024). Research Progress of DDR1 Inhibitors in the Treatment of Multiple Human Diseases. Eur. J. Med. Chem..

[4] Li X., Li Q., Xiong B., Chen H., Wang X., Zhang D. (2022). Discoidin Domain Receptor 1(DDR1) Promote Intestinal Barrier Disruption in Ulcerative Colitis through Tight Junction Proteins Degradation and Epithelium Apoptosis. Pharmacol. Res..

[5] Sanawar R., Sahayasheela V.J., Sarath P., Dan V.M. (2023). Discoidin Domain Receptor 1 Inhibitors: Advances and Future Directions for Novel Therapeutics with Aid of DNA Encoded Library Screens and Artificial Intelligence. Mini Rev. Med. Chem..

[6] Vemula D., Jayasurya P., Sushmitha V., Kumar Y.N., Bhandari V. (2023). CADD, AI and ML in Drug Discovery: A Comprehensive Review. Eur. J. Pharm. Sci..

[7] Virtual Screening Process: A Guide in Modern Drug Designing. https://pubmed.ncbi.nlm.nih.gov/37676591/.

[8] Ferreira L.G., Dos Santos R.N., Oliva G., Andricopulo A.D. (2015). Molecular Docking and Structure-Based Drug Design Strategies. Molecules.

[9] Structure Based Pharmacophore Modeling, Virtual Screening, Molecular Docking and ADMET Approaches for Identification of Natural Anti-Cancer Agents Targeting XIAP Protein. https://pubmed.ncbi.nlm.nih.gov/33603068/.

[10] Marine Natural Products as Source of New Drugs: An Updated Patent Review (July 2018–July 2021). https://pubmed.ncbi.nlm.nih.gov/34872430/.

[11] Molinski T.F., Dalisay D.S., Lievens S.L., Saludes J.P. (2009). Drug Development from Marine Natural Products. Nat. Rev. Drug Discov..

[12] Zhou N., Zheng C., Tan H., Luo L. (2024). Identification of PLK1-PBD Inhibitors from the Library of Marine Natural Products: 3D QSAR Pharmacophore, ADMET, Scaffold Hopping, Molecular Docking, and Molecular Dynamics Study. Mar. Drugs.

[13] Tan H., Li C., Lai T., Luo L. (2023). In Silico Analysis of USP7 Inhibitors Based on Building QSAR Models and Fragment Design for Screening Marine Compound Libraries. Mar. Drugs.

[14] Chen J., Li X., Tao J., Luo L. (2024). Identification of Marine-Derived SLC7A11 Inhibitors: Molecular Docking, Structure-Based Virtual Screening, Cytotoxicity Prediction, and Molecular Dynamics Simulation. Mar. Drugs.

[15] Luo L., Wang Q., Liao Y. (2022). The Inhibitors of CDK4/6 from a Library of Marine Compound Database: A Pharmacophore, ADMET, Molecular Docking and Molecular Dynamics Study. Mar. Drugs.

[16] Qiao X., Wu X., Chen S., Niu M.-M., Hua H., Zhang Y. (2024). Discovery of Novel and Potent Dual-Targeting AXL/HDAC2 Inhibitors for Colorectal Cancer Treatment via Structure-Based Pharmacophore Modelling, Virtual Screening, and Molecular Docking, Molecular Dynamics Simulation Studies, and Biological Evaluation. J. Enzyme Inhib. Med. Chem..

[17] Khedkar S.A., Malde A.K., Coutinho E.C., Srivastava S. (2007). Pharmacophore Modeling in Drug Discovery and Development: An Overview. Med. Chem..

[18] Saikia S., Bordoloi M. (2019). Molecular Docking: Challenges, Advances and Its Use in Drug Discovery Perspective. Curr. Drug Targets.

[19] Sun H., Tawa G., Wallqvist A. (2012). Classification of Scaffold-Hopping Approaches. Drug Discov. Today.

[20] Penzotti J.E., Landrum G.A., Putta S. (2004). Building Predictive ADMET Models for Early Decisions in Drug Discovery. Curr. Opin. Drug Discov. Dev..

[21] Clark D.E., Grootenhuis P.D.J. (2002). Progress in Computational Methods for the Prediction of ADMET Properties. Curr. Opin. Drug Discov. Dev..

[22] Zhang X., Hu Y., Pan Y., Xiong Y., Zhang Y., Han M., Dong K., Song J., Liang H., Ding Z. (2022). DDR1 Promotes Hepatocellular Carcinoma Metastasis through Recruiting PSD4 to ARF6. Oncogene.

[23] Liu X., Li H., Wang T., Yang T., Yang X., Guo K., Hu L., Ming J. (2023). Recombinant Humanized Collagen Type III with High Antitumor Activity Inhibits Breast Cancer Cells Autophagy, Proliferation, and Migration through DDR1. Int. J. Biol. Macromol..

[24] Gonzalez-Molina J., Kirchhof K.M., Rathod B., Moyano-Galceran L., Calvo-Noriega M., Kokaraki G., Bjørkøy A., Ehnman M., Carlson J.W., Lehti K. (2022). Mechanical Confinement and DDR1 Signaling Synergize to Regulate Collagen-Induced Apoptosis in Rhabdomyosarcoma Cells. Adv. Sci..

[25] Su H., Karin M. (2024). Multifaceted Collagen-DDR1 Signaling in Cancer. Trends Cell Biol..

[26] Recent Advances in the Role of Discoidin Domain Receptor Tyrosine Kinase 1 and Discoidin Domain Receptor Tyrosine Kinase 2 in Breast and Ovarian Cancer. https://pubmed.ncbi.nlm.nih.gov/34805157/.

[27] Tremella Fuciformis Polysaccharides Ameliorated Ulcerative Colitis via Inhibiting Inflammation and Enhancing Intestinal Epithelial Barrier Function. https://pubmed.ncbi.nlm.nih.gov/33744251/.

[28] Microplastic Pollution in Seawater and Marine Organisms across the Tropical Eastern Pacific and Galápagos. https://pubmed.ncbi.nlm.nih.gov/33742029/.

[29] Effects of Conditioned Media from Human Umbilical Cord Blood-Derived Mesenchymal Stem Cells in the Skin Immune Response. https://pubmed.ncbi.nlm.nih.gov/33152947/.

[30] Kim H.-G., Tan L., Weisberg E.L., Liu F., Canning P., Choi H.G., Ezell S.A., Wu H., Zhao Z., Wang J. (2013). Discovery of a Potent and Selective DDR1 Receptor Tyrosine Kinase Inhibitor. ACS Chem. Biol..

[31] Rolta R., Yadav R., Salaria D., Trivedi S., Imran M., Sourirajan A., Baumler D.J., Dev K. (2021). In Silico Screening of Hundred Phytocompounds of Ten Medicinal Plants as Potential Inhibitors of Nucleocapsid Phosphoprotein of COVID-19: An Approach to Prevent Virus Assembly. J. Biomol. Struct. Dyn..

[32] Liu B., Zhou J. (2005). SARS-CoV Protease Inhibitors Design Using Virtual Screening Method from Natural Products Libraries. J. Comput. Chem..

[33] Lyu C., Chen T., Qiang B., Liu N., Wang H., Zhang L., Liu Z. (2021). CMNPD: A Comprehensive Marine Natural Products Database towards Facilitating Drug Discovery from the Ocean. Nucleic Acids Res..

[34] Grigoriev I.V., Hayes R.D., Calhoun S., Kamel B., Wang A., Ahrendt S., Dusheyko S., Nikitin R., Mondo S.J., Salamov A. (2021). PhycoCosm, a Comparative Algal Genomics Resource. Nucleic Acids Res..

[35] Afanamol M.S., Dinesh A.D., Ali K.S., Vengamthodi A., Rasheed A. (2023). Drug Repurposing by in Silico Prediction of Cyclizine Derivatives as Antihyperlipemic Agents. In Silico Pharmacol..

[36] Boal Carvalho P., Cotter J. (2017). Mucosal Healing in Ulcerative Colitis: A Comprehensive Review. Drugs.

[37] Li H., Robertson A.D., Jensen J.H. (2005). Very Fast Empirical Prediction and Rationalization of Protein pKa Values. Proteins.

[38] Van Der Spoel D., Lindahl E., Hess B., Groenhof G., Mark A.E., Berendsen H.J.C. (2005). GROMACS: Fast, Flexible, and Free. J Comput. Chem..

[39] Sousa da Silva A.W., Vranken W.F. (2012). ACPYPE—AnteChamber PYthon Parser interfacE. BMC Res. Notes.

[40] Grant B.J., Rodrigues A.P.C., ElSawy K.M., McCammon J.A., Caves L.S.D. (2006). Bio3d: An R Package for the Comparative Analysis of Protein Structures. Bioinformatics.

[41] Kumari R., Kumar R., Lynn A., Open Source Drug Discovery Consortium (2014). G_mmpbsa—A GROMACS Tool for High-Throughput MM-PBSA Calculations. J. Chem. Inf. Model..

[42] Baker N.A., Sept D., Joseph S., Holst M.J., McCammon J.A. (2001). Electrostatics of Nanosystems: Application to Microtubules and the Ribosome. Proc. Natl. Acad. Sci. USA.

