# Supplementary material for: Identification of DDR1 Inhibitors from Marine Compound Library Based on Pharmacophore Model and Scaffold Hopping
Source: Int J Mol Sci. 2025 Jan 27;26(3):1099. doi: 10.3390/ijms26031099 (PMC11817533; doi:10.3390/ijms26031099)
Supplement: Supplementary file 1 [file ijms-26-01099-s001.zip › annex.pdf]

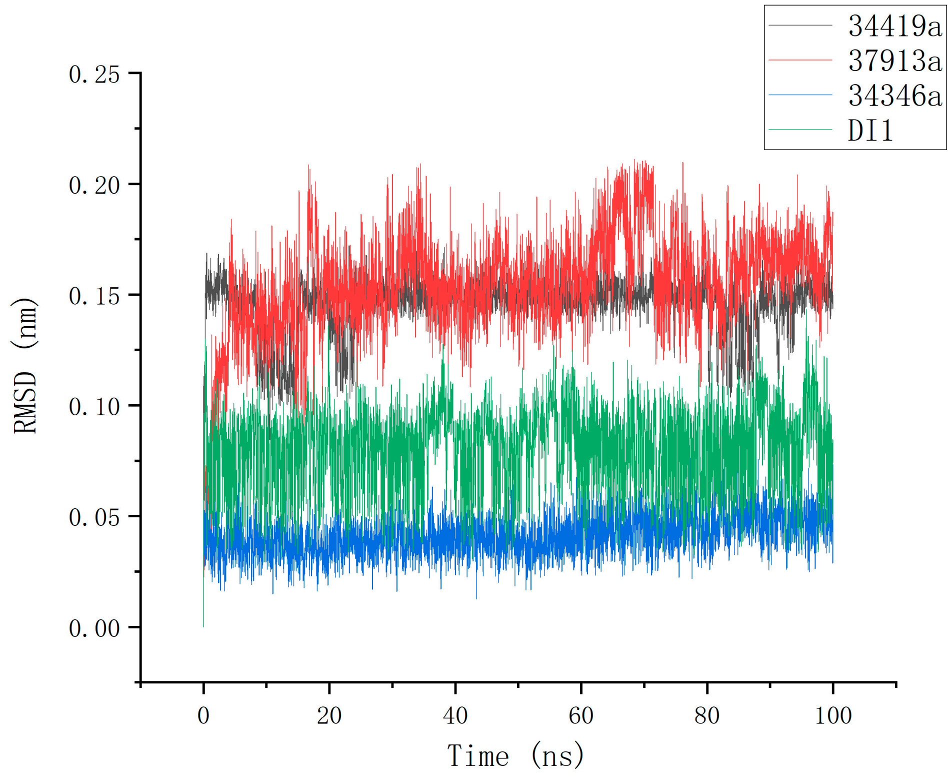

Supplementary Figure S1. RMSD of ligands over time

Supplementary Table S1. pKa and pKmodel of protein amino acids

RESIDUE	pKa	pKmodel
ASP 604A	2.50	3.80
ASP 630A	2.77	3.80
ASP 634A	3.61	3.80
ASP 639	-2.60	3.80
ASP 660A	3.02	3.80
ASP 668A	3.94	3.80
ASP 681A	2.84	3.80
ASP 694A	3.89	3.80
ASP 695A	3.21	3.80
ASP 702A	3.06	3.80
ASP 708A	1.87	3.80
ASP 720	-4.20	3.80
ASP 766A	1.72	3.80
ASP 784A	3.80	3.80
ASP 795A	2.53	3.80
ASP 825A	3.80	3.80
ASP 850A	3.08	3.80
ASP 863A	3.80	3.80
ASP 908A	3.80	3.80

GLU 614A	4.57	4.50
GLU 618A	3.86	4.50
GLU 623A	3.53	4.50
GLU 628A	4.31	4.50
GLU 672A	4.05	4.50
GLU 705A	4.50	4.50
GLU 719A	4.50	4.50
GLU 776A	4.50	4.50
GLU 813A	2.10	4.50
GLU 835A	2.66	4.50
GLU 851A	3.70	4.50
GLU 855A	4.50	4.50
GLU 859A	4.50	4.50
GLU 882A	3.70	4.50
GLU 891A	3.70	4.50
GLU 893A	4.20	4.50
GLU 907A	4.50	4.50
C- 913A	3.20	3.20
HIS 625A	7.09	6.50
HIS 648A	6.36	6.50
HIS 716A	7.45	6.50
HIS 745A	6.50	6.50
HIS 764A	6.50	6.50
HIS 902A	6.50	6.50
CYS 627A	8.64	9.00
CYS 691A	11.55	9.00
CYS 698A	9.23	9.00
CYS 772A	9.00	9.00
CYS 814A	7.69	9.00
CYS 840A	7.40	9.00
CYS 876A	9.00	9.00
CYS 887A	9.00	9.00
TYR 703A	10.62	10.00
TYR 740A	10.00	10.00
TYR 756A	9.65	10.00
TYR 792A	12.93	10.00
TYR 796A	12.09	10.00
TYR 797A	13.40	10.00
TYR 869A	9.86	10.00
TYR 881A	10.00	10.00
N+ 613A	7.93	8.00
LYS 615A	9.94	10.50
LYS 655A	10.29	10.50
LYS 781A	10.36	10.50

ARG 602	12.50	12.50
ARG 607A	11.24	12.50
ARG 609A	11.87	12.50
ARG 611A	11.87	12.50
ARG 645A	12.50	12.50
ARG 658A	12.22	12.50
ARG 666A	12.01	12.50
ARG 678A	12.50	12.50
ARG 686	12.50	12.50
ARG 755A	12.50	12.50
ARG 765A	12.50	12.50
ARG 770A	12.50	12.50
ARG 789A	12.43	12.50
ARG 798A	12.50	12.50
ARG 808A	12.50	12.50
ARG 841A	12.08	12.50
ARG 862A	12.50	12.50
ARG 866A	12.50	12.50
ARG 872A	12.50	12.50
ARG 886A	12.50	12.50
ARG 890A	12.50	12.50
ARG 895A	12.50	12.50
ARG 903A	12.50	12.50
